# Men’s Attitude Towards Contraception and Sexuality, Women’s Empowerment, and Demand Satisfied for Family Planning in India

**DOI:** 10.3389/fsoc.2021.689980

**Published:** 2021-12-16

**Authors:** Iván Mejía-Guevara, Beniamino Cislaghi, Gary L. Darmstadt

**Affiliations:** ^1^ Stanford Aging and Ethnogeriatrics (SAGE) Research Center, Stanford University School of Medicine, Palo Alto, CA, United States; ^2^ Center for Population Health Sciences, Stanford University School of Medicine, Palo Alto, CA, United States; ^3^ Department of Global Health and Development, London School of Hygiene and Tropical Medicine, London, United Kingdom; ^4^ Department of Global Health, Makerere University, Kampala, Uganda; ^5^ Global Center for Gender Equality, Department of Pediatrics, Stanford University School of Medicine, Stanford, CA, United States

**Keywords:** India, men’s attitudinal norms, women’s empowerment, unmet need, contraceptive use, modern methods, female sterilization, district

## Abstract

Whilst the prevalence of unmet need and contraceptive use remained unchanged for 10 years (between 2005–2015) in India, gender restrictive norms and power imbalances also have persisted, preventing married women from meeting their family planning desires. Data for this study are from the 2015–6 National Family Household Survey, which contains information on fertility preferences and family planning for women in reproductive age. As a proxy for men’s attitudinal norms, we aggregated men’s perceptions regarding contraception *(contraception is women’s business, women who use contraception may become promiscuous)* and control over their wife *(if his wife refuses to have sex, men have the right to deny financial support, have sex with another woman, or beat wife)* at district level. Using a three-level random intercepts model, we assessed individual and contextual-level associations of men’s attitudinal norms and met need for contraception among sexually active women (aged 15–49) with any demand for family planning, while adjusting for women’s empowerment indicators [education, job status, and adult marriage] and individual demographic factors. Our results indicate that men’s attitudinal norms are negatively associated with women’s contraceptive use; for instance, a 1 standard deviation increase in the proportion of men who believe that contraception is women’s business was associated with a 12% reduced likelihood of contraceptive use (OR = 0.88, 95% CI 0.82–0.95). Similar associations remained or were stronger after considering only modern methods, or when excluding female sterilization. Furthermore, our contextual effects analysis revealed that women’s higher education or wealth did not improve contraceptive uptake in communities with strong attitudinal norms, but working women or women married as children were more likely to use contraception in those communities. Our results suggest that men’s attitudinal norms may be dominating over women’s empowerment regarding family planning choices among reproductive age women. However, employment appeared to play a strong protective role associated with women’s contraceptive use. It is important for programs seeking to transform gender equality and empower women in making contraceptive choices to consider women’s employment opportunities and to also address male attitudinal norms in the context of the ecosystem in which men and women coexist and interact.

## Introduction

In India, the burden of family planning still falls on women due to persistent gender restrictive norms and inequalities, as well as kinship structures and other cultural contextual factors (Malhotra, Vanneman, and Kishor 1995; Seth et al., 2020). These gender-related socio-cultural factors have contributed to the stagnation observed in the prevalence of modern contraceptive use and unmet need for family planning—around 50 and 14 percent, respectively—during 2005 and 2015 (IIPS and ICF 2017; S. K. Singh et al., 2019). The extent to which women’s empowerment is an enabling factor to offset the pervasive role of norms that reflect gender inequalities in contraceptive access and utilization has not been fully addressed.

Few studies have investigated the role of men and community-level factors—particularly with regard to the persistence of cultural factors, traditional family roles, gender egalitarian values, and patriarchal gender order—in India and other regions, which tend to undermine women’s equality and empowerment (Schensul et al., 2015; Mishra et al., 2014; Gruber and Szołtysek 2016; Cislaghi et al., 2020; A. Singh et al., 2021). For instance, patriarchy is “a system of society or government in which men hold the power and women are largely excluded from it”^1^, with implications on women’s decision making, well-being, and health (Jewkes and Morrell 2018; L. Heise et al., 2019; Rivera and Scholar 2020). Further, anthropological studies highlight the complexity of men’s intervention in women’s reproductive health, the importance of particular cultural contexts, and the distinction between equality and equity in reproductive health services (Dudgeon and Inhorn 2004). There is substantive evidence behind the pervasive effects of environments of inequality and control that undermine women’s ability to make reproductive health and family planning decisions. For instance, patriarchal masculinities that emphasize the superiority of the authority of men over women are regarded as important predictors of domestic violence (Jewkes and Morrell 2018; Sikweyiya et al., 2020) and are key aspects of the cultural normative and social environment which shape relations and power dynamics between men and women (Suzuki et al., 2011; Mshweshwe 2020).

Here we emphasize the potential role of women’s equality as a precondition for securing wellbeing and prosperity for populations, and its catalytic effect on contraceptive provision and use (Slaymaker et al., 2020). To study how to overcome gender inequalities or to gain greater reproductive autonomy in restricted environments with high fertility prevalence, some studies have examined programmatic approaches to renegotiate gender relationships with promising evidence in a Mumbai slum (Cislaghi et al., 2020), or the covert use of contraception among women in need of family planning in sub-Saharan Africa (SSA), where contraceptive use has been positively associated with working outside the home but negatively related to more years of schooling (Gasca and Becker 2018; OlaOlorun, Anglewicz, and Moreau 2020). Women’s self-help groups (SHGs) is another mechanism associated with a significant impact on women’s economic, social, and political empowerment in South Asia and other developing countries (Brody et al., 2017), although still with limited or no impact on psychological empowerment or on attitudes towards domestic violence and respect within households in India (Kumar et al., 2021).

Women’s empowerment has been regarded as a fundamental instrument to reduce gender inequalities. It is characterized as the promotion of “women’s sense of self-worth, their ability to determine their own choices, and their right to influence social change for themselves and others” (World Vision 2021). It has also been positively associated with aspects of maternal and child health, including exposure to violence and contraception use, among others (Pratley 2016). Oher studies indicate that the relationship between women’s empowerment and family planning is mixed and depends on the empowerment domain and family planning outcome investigated (Prata et al., 2017; Yaya et al., 2018). Further, establishing that interventions lead to empowerment of women and women’s empowerment leads to changes in family planning practices remains challenging (Mandal, Muralidharan, and Pappa 2017), or less explored, and reverse effects have also been found in India where higher contraceptive use leads to advances in women’s empowerment (Dhak, Saggurti, and Ram 2020). Education, access to cash or employment^2^, and household decision making are commonly used indicators of women’s empowerment or economic participation (L.L. Heise and Kotsadam 2015) and related to family planning (Yaya et al., 2018). In India, there is evidence of positive associations of contraceptive use among uneducated women (McNay, Arokiasamy, and Cassen 2003)—for those with higher household decision making and who are currently working–but also of mixed affects when investigating other domains of empowerment (Reed et al., 2016; S. K. Singh et al., 2019). In addition, women’s empowerment approaches that promote economic decision making, negotiation of sexual activity, or perceived agreement on fertility preferences were also related to higher contraception use in selected African countries (Do and Kurimoto 2012). Women’s empowerment was also related to increase in attitudes for safer sex negotiation in several countries from South Asia and SSA, although the association vary across countries and age or other demographic characteristics (Tenkorang 2012; Atteraya, Kimm, and Song 2014; Jesmin and Cready 2014; Sano et al., 2018; Asabu 2021; Putra, Dendup, and Januraga 2021).

In this study we aim to examine the extent to which men’s attitudinal norms towards contraception and sexuality influence women’s ability to satisfy their demand for contraception, and whether various forms of women’s empowerment can neutralize or offset potential negative effects of those norms. We hypothesized that distinct domains of women’s empowerment may have limited effects on women’s contraceptive uptake in contexts with strong or prevalent restrictive norms. Here we measured men’s attitudinal norms as community-level aggregates of individual men’s attitudes and beliefs regarding women’s contraceptive use and control of their wives. We approximated women’s empowerment using community-level indicators of education, labor, and adult (as opposed to child) marriage, building on evidence from previous studies (Raj et al., 2009; L. L. Heise and Kotsadam 2015; Mejía-Guevara et al., 2020). We used data from the latest wave of the National Family Health Survey (NFHS-4^3^), and followed a multilevel approach as this dataset provides representative estimates at the district level, and it is the most general framework for the assessment of contextual effects and geographic heterogeneity (Diez-Roux 1998; Bell and Jones 2015; Mejía-Guevara et al., 2018). Finally, our analytical approach is designed to test the extent to which the distinct domains of women’s empowerment can offset any potential effect of men’s attitudinal norms across Indian communities. We hypothesized that use of this approach would shed new light on the interaction between gender restrictive norms and women’s empowerment and could help to inform policies to increase gender equality in India.

## Materials and Methods

### Data and Study Population

Data for this research came from the NFHS-4 for 2015–16, which is a nationally representative survey from India that provides comprehensive data on fertility preferences, family planning, and other demographic characteristics and health outcomes for women in reproductive ages 15–49 and their under-5 year-old children. Previous waves of the NFHS provided representative information at the state and rural/urban levels, but the NFHS-4 is the first wave designed to additionally provide representative data at the district level. NFHS-4 is a stratified two-stage sample, with primary sampling units (PSUs) selected in the first stage from 2011 census data that served as a sampling frame comprised of villages in rural areas and Census Enumeration Blocks (CEBs) in urban areas. In the first stage, PSUs in rural and urban strata were selected from the sampling frame with probability proportional to sampling size (PPSs). In the second stage, households were selected systematically from clusters of approximately 100–150 households defined by previously selected PSUs or segmented PSUs. Additional details of the sampling survey design are available elsewhere (IIPS and ICF 2017). NFHS-4 contains anonymous, publicly available data with no personally identifiable information. NFHS-4 protocol was approved by the Institutional Review Boards of IIPS and ICF International.

### Participants

**FIGURE 1:**
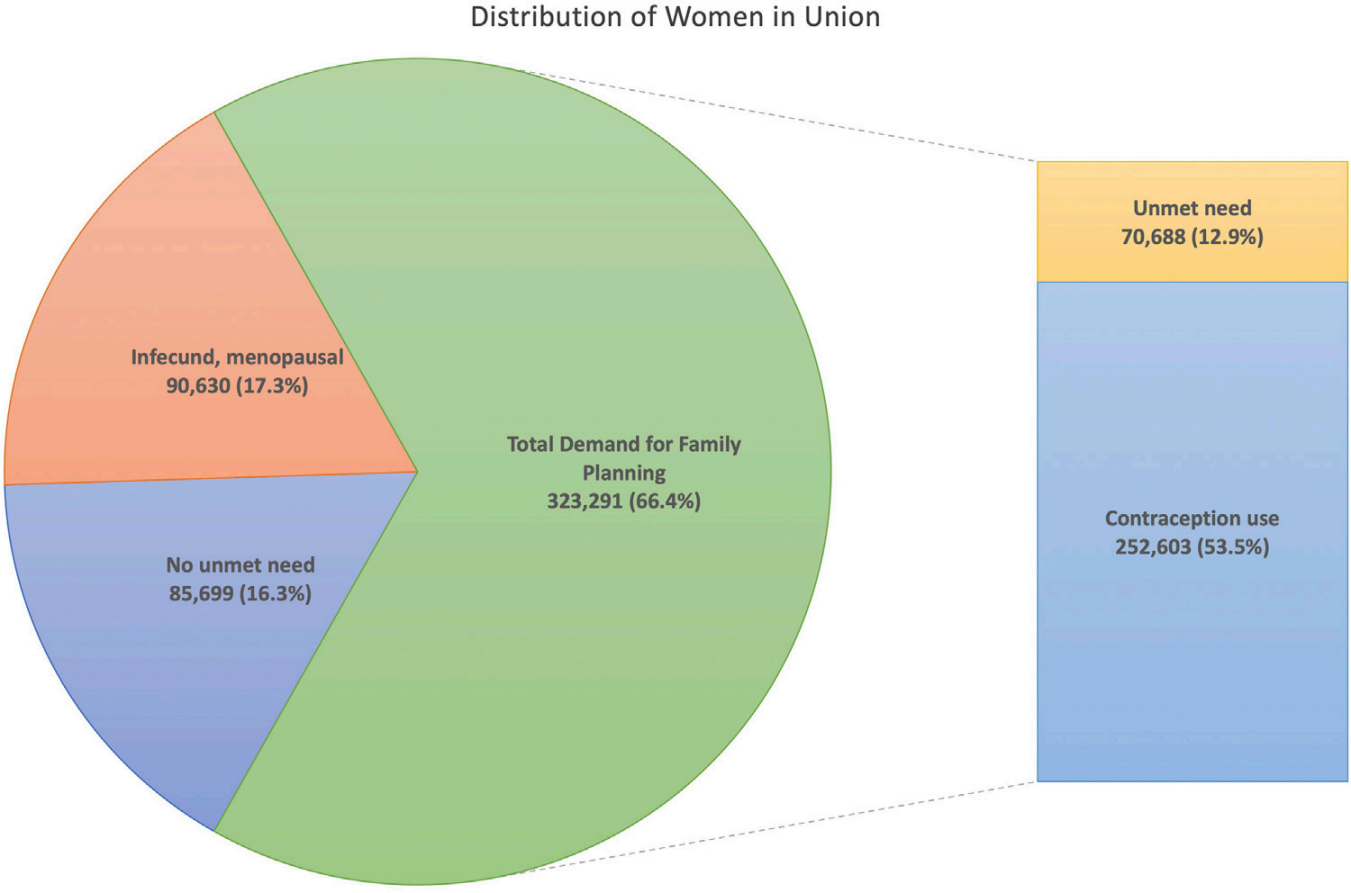
Use and demand for family planning among sexually active married women aged 15–49 in India, NFHS-4 (2015–2016). Note: We excluded seven women with missing information.

The NFHS-4 is part of the Demographic and Health Surveys (DHS) Program and contains three core questionnaires: a household questionnaire, a men’s questionnaire, and a women’s questionnaire. For this study, we used information from the men’s and women’s questionnaires. The former includes a sample of 112,122 men respondents aged 15–54, that we used to construct community-level exposures reflecting gender egalitarian attitudes related to contraception and control of women. The latter is comprised of 699,686 women respondents aged 15–49 that we used to construct community- and individual-level indicators of women’s empowerment as well as individual covariates. However, for statistical analysis, we only considered a sample of 499,627 women who were married or in union because our primary interest was on sexually active women whose family planning decisions may have been affected by their daily interactions with men (Table 1). Among those married or in union, we further restricted our sample to a subgroup of 323,291 (66.4%) women with any demand for family planning^4^, after we excluded 90,630 (17.3%) infecund or menopausal women, 85,699 (16.3%) women with no unmet need—i.e., women who have or recently had an intended pregnancy or want to have a birth in less than 2 years—and seven women who declined to answer (0.001%) (Figure 1). Finally, for fully adjusted multilevel analysis, our sample size was further reduced to 57,341 because that is the number of women who reported their job status, a key indicator for the assessment of women’s empowerment.

**TABLE 1:** Total demand for family planning for married women aged 15–49: unmet + met contraception need by type of method, NFHS-4.

	Modern				
	No method	Traditional	Female Sterilization	Other	Total
Unmet need					
for spacing	32094 (8.5)				32094 (8.5)
for limiting	38594 (10.9)				38594 (10.9)
Contraception use					
for spacing	9144 (2.5)		1 (0.0)	20819 (5.8)	29964 (8.2)
for limiting	22609 (6.4)		157610 (54.2)	42420 (11.9)	222639 (72.4)
Total demand for family planning	70688 (19.4)	31753 (8.8)	157611 (54.2)	63239 (17.6)	323291 (100)

Total observations in sample, weighted proportions in parentheses.

### Outcome

The main outcome of this study is the use of contraception—or demand satisfied—for women with any demand for family planning. In our baseline model (Model 1), we included any method for contraception, but we also excluded traditional/folkloric methods and perform sensitivity analysis with modern methods only (Model 2). Traditional/folkloric methods include period abstinence, or withdrawal, while modern methods include pills, intrauterine devices (IUDs), injections, diaphragms, female/male condoms, female/male sterilization, lactational amenorrhea, or foam and jelly^5^. Finally, since female sterilization has been reported as a highly prevalent method of contraception in India (IIPS and ICF 2017), we also conducted sensitivity analysis by excluding this type of method (Model 3).

### Community Gender Norms Exposures

#### Men’s Attitudinal Norms.

Our analysis relies on information from 640 districts. A district is a rural/urban administrative division of an Indian state or territory that is operationalized as a community for this study because it was the lowest administrative level available in NFHS-4. Gender norms were approximated as ‘collective attitudinal norms’ (L. L. Heise and Kotsadam 2015; Chung and Rimal 2016; Weber et al., 2019) using urban/rural district-level aggregates of men’s attitudes towards contraceptive use and beliefs about control of their wife. We tested five specific indicators for our assessment of men’s attitudinal norms, using the following items available from the NFHS-4: 1) Contraception is woman’s business and a man should not worry; 2) Women who use contraception may become promiscuous; if wife refuses to have sex, husband has the right to: 3) refuse financial support, 4) have sex with another woman, and 5) beat wife. In addition, similar to previous studies (Mishra et al., 2014), we constructed a composite index as a mix of these five indicators—as a summation of men’s responses to these five items—to measure the combined effect of women’s exposure to one or multiple of men’s restrictive norms. For simplicity, hereinafter we refer to this indicator as the Male Attitudinal Scale, and it varies from 0 (if man responded negatively to all items) to 5 (if man responded positively to all items).

#### Women’s Empowerment.

To measure the extent to which individual and community-level effects of women’s empowerment are able to offset men’s attitudinal norms, we constructed and tested three indicators, defined as: 1) completing secondary education, 2) adult marriage, and 3) currently employed^6^ (L. L. Heise and Kotsadam 2015). They were constructed as district-level aggregates of the respective individual responses for women aged 15–49 in the district for whom complete data was available.

### Covariates

In our statistical analysis we accounted for factors identified in previous studies as related to family planning decisions (Raj et al., 2009; S. K. Singh et al., 2019; Mejía-Guevara et al., 2020), including age, parity, religion, place of residency, wealth status, as well as individual indicators of women’s empowerment, including education, adult marriage, and job status. Age was measured in single years and specified as continuous for regression analysis; parity was coded in five categories as 0, 1, 2, 3, and 4 or more children; religion was coded in seven group categories: Hindu, Muslim, Christian, Sikh, Buddhist, Other, and No religion; and place of residency was coded as urban or rural. We used the wealth status constructed by the NFHS as a wealth index reflecting a household’s cumulative living standard and categorized in quintiles^7^ (Rutstein and Johnson 2004). Education was coded in four categories: no education (0 years), primary (0–5 years), incomplete secondary (6–11 years), and complete secondary or higher (12 + years). Finally, adult marriage—defined as marriage or informal union at age 18 or older—and job status were binary coded. For job status we included both women who worked for pay (80%, cash or in-kind) and those who did not, after conducting sensitivity analysis that excluded the latter group and which did not change our findings (not shown).

### Statistical Methods

We assessed the association between community gender norms and use of contraception using a three-level random intercept Multilevel Logistic model. The model specification is as follows:

$$y_{ijk}=\beta_{0}+\beta_{1,jk}Z_{jk}+\beta_{2,jk}X_{ijk}+v_{k}+\mu_{jk},$$ (1)

where $y_{ijk}$ represents the individual-level use of contraception of a woman *i*, in district *j*, in state *k*. $\;Z_{jk}$ is a vector containing a male attitudinal norm exposure variable and indicators reflecting women’s empowerment, all measured at the district level *j* in each state *k*. $X_{ijk}$ represents a vector of individual level exposures and covariates for each woman at the respective three levels (i.e., individual, district, state). Finally, the terms $\mu_{jk}$ , $v_{k}$ are random coefficients that represent the residual variation at the district and state level, respectively. The model was fitted using maximum likelihood methods. Hereinafter we refer to this model as baseline or Model 1. Models that only consider modern contraception in the outcome are referred to as Model 2, and models that further exclude female sterilization as Model 3.

Of particular interest for this study is to examine the extent to which women’s empowerment and wealth status can protect women from adverse environmental influences characterized by strong attitudinal norms in the communities in which they reside. We modeled these contextual effects by introducing an interaction term in Eq. 1, $x_{ijk\;}$ * $z_{jk}$ ( $x_{ijk\;}\in X_{ijk},\;z_{jk}\in Z_{jk}$ ), where $x_{ijk\;}$ stands for the individual-level variable representing women’s empowerment (e.g., education), and $z_{jk}$ is the compositive index of male attitudinal norms at the community level. We then fitted four separate models to measure the effect of each women’s empowerment variable (education, adult marriage, and job status) and wealth status on varying values of the Male Attitudinal Scale.

Finally, to account for potential heterogenous effects of regional disparities, we conducted stratified analysis for six different regions of India: Central, East, North, Northeast, South, and West, which are comprised of 29 states and seven union territories^8^. Statistical analysis was conducted using Stata^©^ 16.1 (StataCorp LLC, College Station, TX).

## Findings

### Descriptive Analysis

In India, 19.4% of married women with any demand for family planning had unmet need—8.5% for spacing and 10.9% for limiting the number of children. Among the other 80.6% of women who reported using contraception, the large majority were using modern contraceptives (71.8%), with 54.2% of them using female sterilization, as opposed to traditional/folkloric methods (8.8%). Most women used contraception to limit (72.4%) rather than space (8.2%) pregnancies (Table 1).

Table 2 shows the numbers and percentages of men who responded positively to the set of five indicators reflecting attitudinal norms towards contraception and sexuality. The most prevalent norms expressed by 36.1% of men was the attitude that contraception use is women’s business, followed by the belief that women who use contraception are promiscuous (19.8%). The least prevalent was the belief that a man has the right to beat his wife if she refuses to have sex (8.2%). The combined prevalence of these five indicators—as measured by the Male Attitudinal Scale—indicates that 51.2% of men responded in favor of at least one attitudinal norm, with 29.5% expressing only one and 15.6% expressing two of them. Table 2 also shows that 21.5% of women completed at least secondary education, 56.2% got married at age 18 or older, and 24.0% were currently working.

**TABLE 2:** Sample size, and attitudinal (men aged 15–54) and empowerment (women aged 15–49) norms at the district level, NFHS-4.

Gender norms exposures	N (weighted %)
Men’s attitudinal norms indicators	
M1. Contraception is woman’s business—man should not worry	39350 (36.1)
M2. Women who use contraception become promiscuous	22059 (19.8)
M3. If wife refuses to have sex, husband has the right to refuse financial support	10490 (9.4)
M4. Husband has the right to have sex with other women if wife refuses to have sex	9425 (8.4)
M5. Beating justified if wife refuses to have sex with husband	9102 (8.2)
Male Attitudinal Scale (MAS)	
0	54124 (48.8)
1	32686 (29.5)
2	17305 (15.6)
3	4698 (4.2)
4	1664 (1.5)
5	476 (0.4)
Women’s empowerment indicators	
W1. Complete secondary or higher education	140979 (21.5)
W2. Adult marriage	290071 (56.2)
W3. Currently working	28636 (24.0)

For robustness, district -level percentages were obtained after further excluding districts with less than 20 observations, which were comprised of 1,169 individuals in the male sample and 37 individuals in the female sample.

**FIGURE 2:**
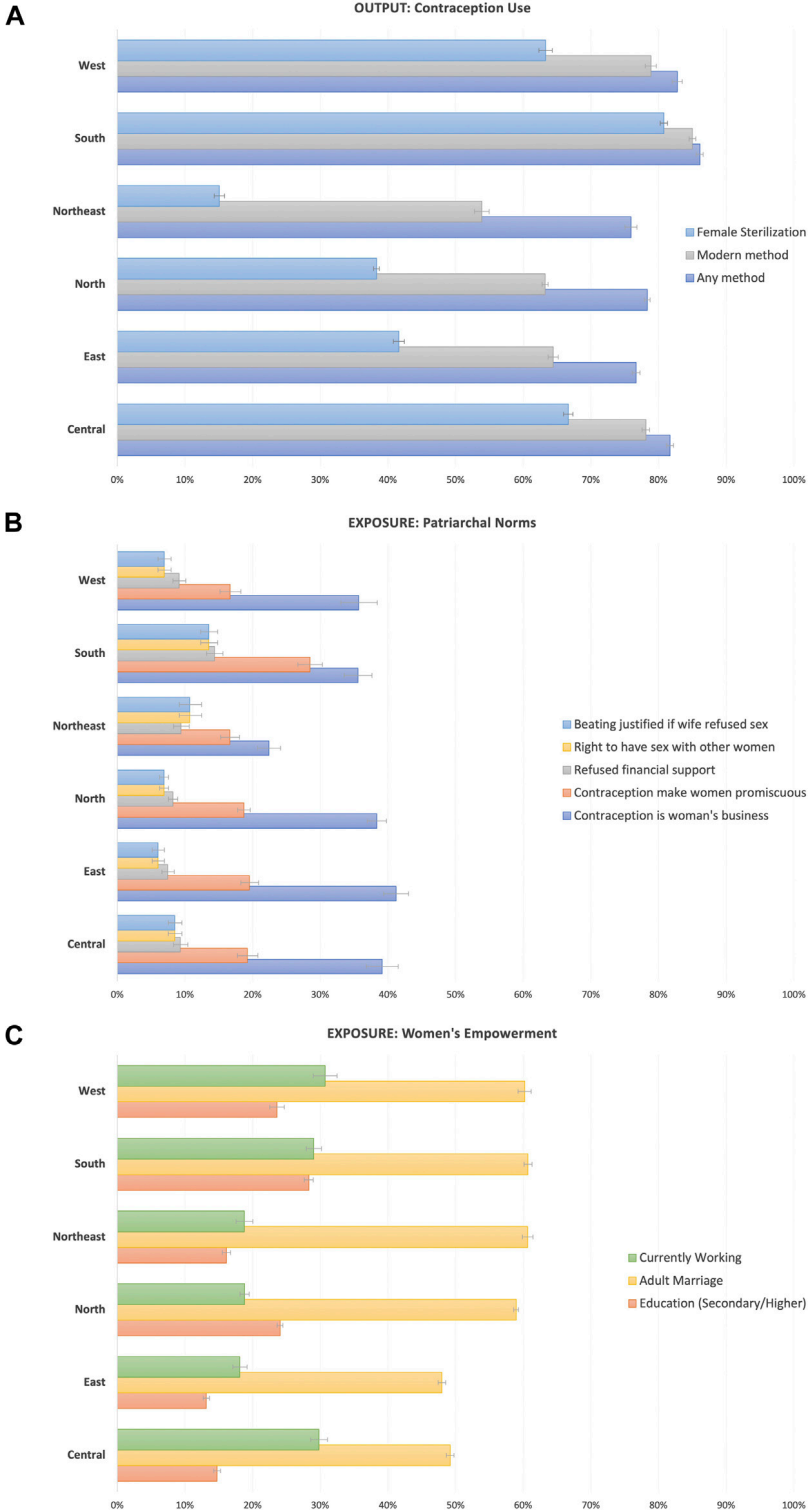
Regional-level percentage of women using contraception **(A)**, men’s attitudinal norms **(B)**, and women’s empowerment **(C)**. Note: For robustness, district -level percentages were obtained after further excluding districts with less than 20 observations, which were comprised of 1,169 individuals in the male sample and 37 individuals in the female sample.

A regional descriptive assessment of contraceptive use revealed that high percentages of women had satisfied their demand for contraception with any method, varying from 76% in the Northeast region to 86% in the South. However, those differences widened when only considering modern methods, where the demand satisfied in the Northeast dropped to 54%, while it was unchanged in the South (85%). We also observed important differences in the prevalence of traditional vs. modern methods in the Northeastern and Eastern regions, where there was relatively more use of traditional methods than in other regions (Figure 2A). Regarding men’s attitudinal norms, the attitude that contraception is women’s business was the most prevalent indicator—although with important differences in magnitude—across all regions, varying from 22% in the Northeast to 41% in the East. Important differences across regions can also be observed in the belief that women who use contraception become promiscuous (Figure 2B). Finally, we appreciate noticeable regional differences in indicators of women’s empowerment, with low percentages of women who were educated (varying from 13% in the East to 28% in the South) or in the labor force (from 18% in the East and North to 31% in the West), but larger percentages of women who were married at age 18 or older (from 48% in the East to 63% in the North (Figure 2C).

### Multi-Level Analysis

**FIGURE 3:**
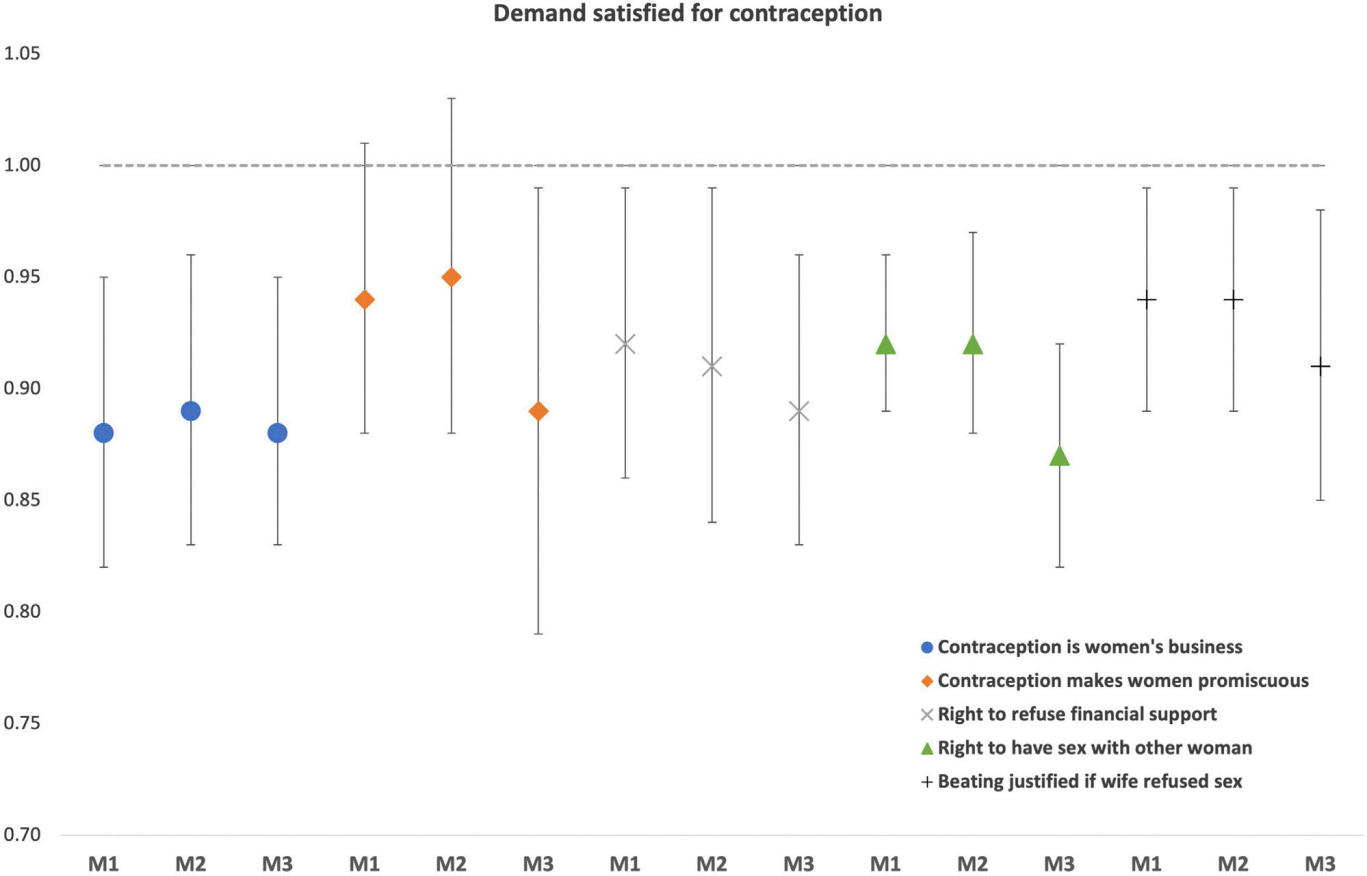
Multilevel associations between men’s attitudinal norms and use of contraception: any method (Model 1: M1), modern method (Model 2: M2), and modern method excluding female sterilization (Model 3: M3), NFHS-4. Note: Results are from separate fully-adjusted models: baseline (M1), modern methods (M2), and modern methods but excluding female sterilization (M3), where we accounted for women’s empowerment indicators at the individual and district levels, as well as for demographic indicators at the individual level.

Our baseline fully adjusted multilevel model revealed that all men’s attitudinal norms were negatively associated with the use of contraception, with slight differences in effect size across most indicators. The strongest effect was for the norm reflecting contraception as women’s business, where one standard deviation increase in the community prevalence of this indicator had the lowest odds (Odds Ratio [OR] = 0.88; 95% CI 0.82–0.95) for demand satisfied with any method compared with the effect from other attitudinal norm indicators, for which the ORs ranged between 0.92–0.94 (Model 1 in Figure 3). We replicated the same analysis but including only modern methods in our outcome and found no significant changes compared to the baseline model (Model 2 in Figure 3). After further excluding female sterilization as a modern method for sensitivity as it is by far the most popular contraceptive method in India (Model 3), we found stronger negative effects of attitudinal norms on contraceptive use, although the associations were statistically different only for the belief that contraception makes women promiscuous (0.89; 0.79–0.99) and for the belief that the husband has the right to have sex with another woman if his wife refuses to have sex with him (0.87; 0.82–0.92) (Model 3 in Figure 3).

Our previous results remained consistent when using the Male Attitudinal Scale as the main exposure, reflecting the combined effect of the five gender norms indicators. That is, a 1 SD increase in the Male Attitudinal Scale was associated with lower odds of contraception use, with a stronger effect when excluding female sterilization. The respective results for each model were (Table 3): Model 1 (OR = 0.86; 95% CI: 0.80–0.92), Model 2 (0.87; 0.80–0.93), and Model 3 (0.82; 0.76–0.90).

**TABLE 3:** Multilevel associations of men’s attitudinal norms and demand satisfied for contraception, India NFHS-4.

	Model 1:	Model 2:	Model 3:
	Demand satisfied with any method	Demand satisfied with modern methods	Demand satisfied with modern methods - excluding female sterilization
	Odds ratio and 95% CI (except for Random Coefficients)		
District level Exposures			
Male Attitudinal Scale (MAS: 1 SD)	0.86	0.87	0.82
	(0.80–0.92)	(0.80–0.93)	(0.76–0.90)
Women’s Empowerment (1 SD)			
Complete secondary/higher	1.12	1.12	0.99
	(0.96–1.31)	(0.96–1.31)	(0.82–1.20)
Adult marriage	0.90	0.91	1.07
	(0.75–1.09)	(0.74–1.11)	(0.83–1.39)
Currently working	1.07	1.06	1.00
	(0.99–1.16)	(0.97–1.16)	(0.90–1.10)
Individual Exposures			
Education (Ref: secondary/higher)			
No education	1.19	1.18	0.60
	(1.00–1.41)	(0.97–1.43)	(0.48–0.75)
Primary	1.24	1.25	0.77
	(1.07–1.44)	(1.06–1.48)	(0.60–0.98)
Incomplete secondary	1.14	1.12	0.83
	(1.00–1.29)	(0.97–1.31)	(0.72–0.94)
Child marriage (Ref: adult marriage)	1.60	1.64	1.18
	(1.48–1.73)	(1.53–1.76)	(1.08–1.28)
Currently working (Ref: not working)	1.33	1.34	1.26
	(1.23–1.43)	(1.24–1.44)	(1.11–1.44)
Random Coefficients			
Variance: State	0.46	0.52	0.83
	(0.29–0.74)	(0.28–0.95)	(0.56–1.24)
Variance: District	0.29	0.29	0.34
	(0.20–0.43)	(0.20–0.42)	(0.24–0.49)
Observations	56,549	50,783	23,193
Number of groups	36	36	36

For Model 1 the outcome includes any method of contraception, for Model 2 the outcome only includes modern methods, and for Model 3 female sterilization is further excluded. Results are from fully-adjusted models, where we additionally accounted for individual covariates (age, parity, religion, household wealth, and place of residency).

All these models were robust to the combined effects of women’s empowerment at the individual and district level, as well as to individual level covariates. For instance, the odds of contraceptive use—conditional on men’s attitudinal norms—increased with 1 SD increases in the percentage of women who were educated (1.12; 0.96–1.31) and currently working (1.07; 0.99–1.16), and increased for child marriage (younger than 18 years old) (1.60; 1.48–1.73), although they were marginally or not associated (Table 3). However, these women’s empowerment indicators were statistically significantly associated with increased contraceptive use when tested at the individual level, with stronger effects seen for more disadvantaged groups in the case of education [ORs 1.19 (1.00–1.41) for women with no education relative to educated women] and marriage [OR 1.60 (1.48–1.73) for women who got married when they were children relative to those married during adulthood]. In contrast, the odds of contraceptive use were higher [OR 1.33 (1.23–1.43)] for currently working women relative to those out of the labor force.

### Contextual Effects

**FIGURE 4:**
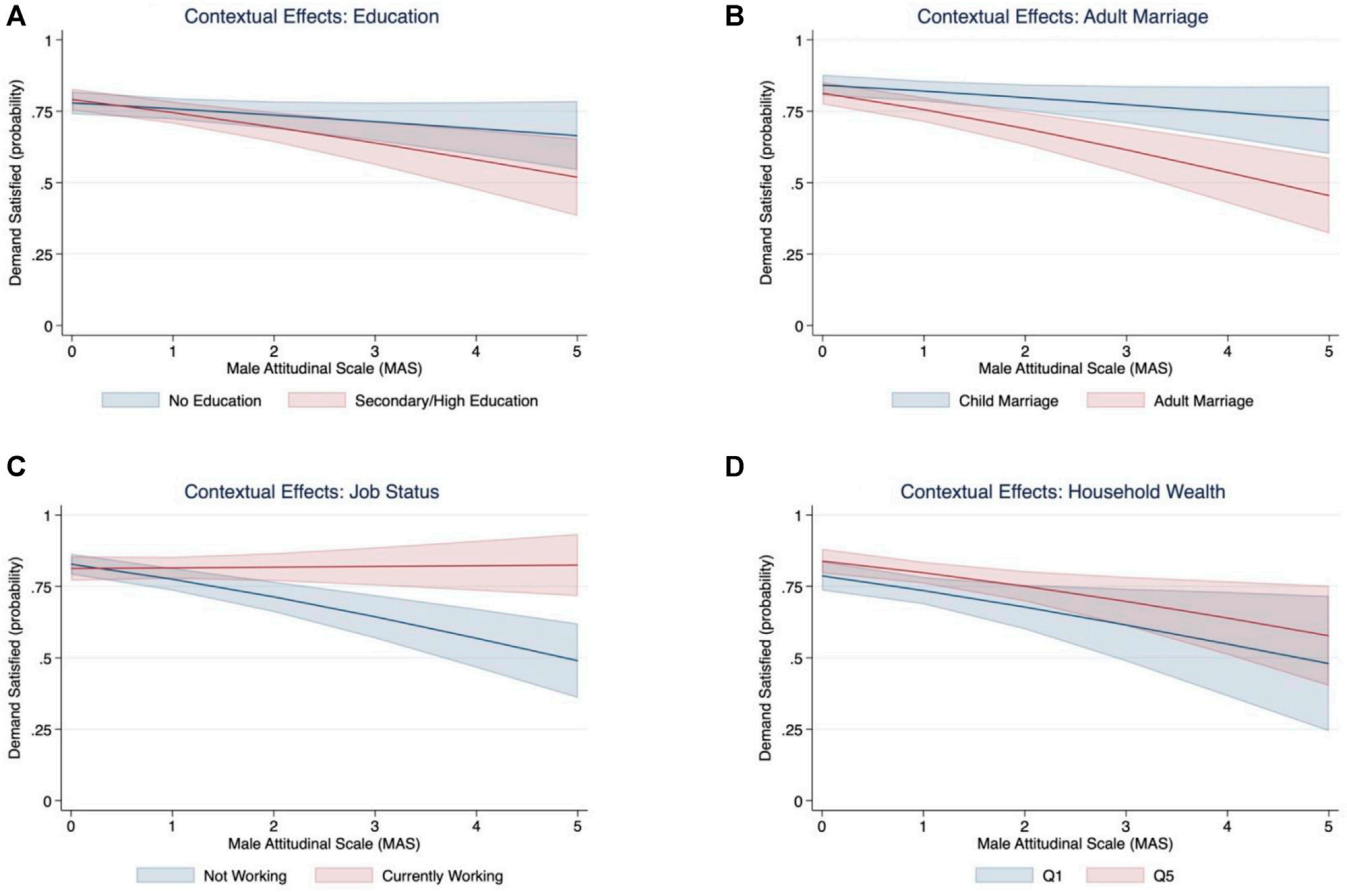
Contextual effects associations between men’s attitudinal norms and women’s empowerment (**(A)** Education, **(B)** Adult Marriage, **(C)** Job Status), and Household Wealth **(D)**. Note: Q1 and Q5 stand for the wealth quintiles 1 and 5, respectively.

We show here results of the contextual effects of women’s empowerment in settings with varying levels of acceptance of male attitudinal norms (Figure 4). We found that increases in the prevalence of attitudinal norms—as measured by increasing values of the Male Attitudinal Scale indicator—were negatively associated with a lower probability of contraceptive use, regardless of individual levels of women’s education (Figure 4A) or wealth (Figure 4D). In the case of education, women with higher levels of education were less likely to use contraception in districts with strong attitudinal norms compared with less educated women, although the interaction was not statistically robust (overlapping confidence intervals in Figure 4A). A separate model revealed that women who get married as adults were less likely (50%) to use contraception than women who experienced child marriage (75%), and the difference was statistically robust (Figure 4B). Finally, jobs had protective effects on the use of contraception, as currently employed women were significantly more likely to use contraception than unemployed women, even in communities with a higher exposure to restrictive attitudinal norms (Figure 4C).

### Regional Analysis

We conducted regional analysis as a check for robustness of findings. The multilevel association between men’s attitudinal and women’s empowerment norms remained consistent across most regions (Table 4 **)**. Effect sizes and significance levels varied across regions, particularly for the North and Northeast areas, where some indicators showed opposite associations, although these were not statistically significant.

**TABLE 4:** Multilevel associations of attitudinal and empowerment norms and demand satisfied for contraception, by Indian Region (NFHS-4).

	Central	East	North	Northeast	South	West
	Odds ratio and 95% CI (except for Random Coefficients)					
Men’s Attitudinal Norms (1 SD)						
Male Attitudinal Scale (MAS)	0.87	0.81	0.84	1.03	0.97	0.77
	(0.81–0.94)	(0.65–1.02)	(0.79–0.90)	(0.95–1.11)	(0.93–1.01)	(0.62–0.96)
Contraception is women’s business	0.93	0.87	0.88		0.96	0.83
	(0.91–0.94)	(0.73–1.03)	(0.80–0.97)		(0.83–1.12)	(0.61–1.12)
Contraception makes women promiscuous	0.90	0.96	0.92		0.98	1.02
	(0.81–1.00)	(0.81–1.14)	(0.87–0.97)		(0.88–1.08)	(0.68–1.54)
Husband right to refuse financial support	0.92	0.91	0.87		1.02	0.81
	(0.79–1.06)	(0.77–1.07)	(0.78–0.97)		(0.96–1.09)	(0.55–1.20)
Husband right to have sex with another woman	0.96	0.86	0.87		0.99	0.81
	(0.86–1.07)	(0.62–1.20)	(0.80–0.95)		(0.96–1.02)	(0.66–0.98)
Beating justified if refused sex	0.89	0.93	0.98		0.98	0.85
	(0.78–1.02)	(0.82–1.05)	(0.89–1.08)		(0.91–1.05)	(0.83–0.88)
Women’s Empowerment (1 SD)^a^						
Complete secondary/higher	0.87	1.09	0.98	0.67	1.29	1.32
	(0.68–1.11)	(0.64–1.85)	(0.74–1.30)	(0.48–0.94)	(1.05–1.58)	(1.17–1.49)
Adult marriage	1.28	0.78	1.14	1.04	0.74	0.74
	(0.95–1.72)	(0.60–1.03)	(0.80–1.63)	(0.75–1.45)	(0.54–1.02)	(0.66–0.82)
Currently working	1.11	1.03	0.96	0.66	1.03	1.19
	(0.99–1.26)	(0.89–1.19)	(0.86–1.06)	(0.61–0.71)	(0.86–1.22)	(1.15–1.23)
Random Coefficients						
Variance: State	0.03	0.94	0.18		0.04	0.23
	(0.02–0.06)	(0.36–2.41)	(0.07–0.46)		(0–1.08)	(0.13–0.42)
Variance: District	0.38	0.28	0.19	0.06	0.28	0.21
	(0.19–0.77)	(0.16–0.48)	(0.06–0.58)	(0.02–0.20)	(0.19–0.40)	(0.16–0.27)
Observations	13,421	9,250	13,571	6,296	8,176	5,815
Number of groups	3	4	8	8	8	5

Results are from fully-adjusted models, where we additionally accounted for individual empowerment indicators (education, adult marriage, and job status), as well as for individual covariates (age, parity, religion, household wealth, and place of residency).

a Results from these models are from the model using the Male Attitudinal Scale as main exposure.

## Discussion

In this paper, we had an interest to understand the extent to which gender norms could affect women’s capacity to meet their family planning needs. Our study generated four salient results related to its aim. First, a large percentage of married women in India had a demand for family planning (66.4%), and most of them (∼70%) used contraception and preferred modern rather than traditional methods. In addition, female sterilization remained the most popular method among women with demand for family planning (54.2%). Second, men’s attitudinal norms in India were prevalent, particularly the attitude that contraception is women’s business and men should not worry about it (36%). At the same time, while a large percentage of women (56%) reported being married at an adult age, less than 30% have completed secondary/higher education or were currently working. Third, we found evidence that men’s attitudinal norms were negatively associated with demand satisfied for contraception, and the evidence remained robust when we restricted the sample to those women only using modern methods and became stronger after excluding female sterilization. Fourth, women’s empowerment indicators were limited in offsetting the pervasive effects of men’s attitudinal norms on contraception use, particularly in communities where those norms were the most dominant, except that currently working was a protective factor in all types of communities.

Recent studies have documented that levels of unmet need and contraceptive use have been stagnated for at least 10 years in India (IIPS and ICF 2017; S. K. Singh et al., 2019). Our analysis from using the most recent data from the NFHS-4 revealed regional differences in contraceptive uptake, with Central, West, and South regions having the highest prevalence, but mostly explained by the use of modern methods, compared to Northeastern and Northern regions, where female sterilization is relatively less popular and traditional methods are relatively more popular than in other regions, but modern methods still predominate. Female sterilization is by far the dominant method in those regions with higher uptake of modern contraception. Although a better understanding of the reasons for Indian women to heavily rely on female sterilization is out of the scope of this study, it is worth pointing out that regretting sterilization is on the rise, particularly for women from low-fertility regions compared to regions with high fertility and following the loss of a child (S. K. Singh et al., 2019). Sterilization in those regions is associated with cultural factors (Saavala, 1999), as it is seen by women in the South as a way to gain greater autonomy—e.g., decision-making for household purchases and freedom of mobility—once they have completed their childbearing. Sterilization is also increasing over time for younger and lower parity women, as it accelerates the acquisition of autonomy by altering power dynamics between mothers-in-law and daughters-in-law in settings where most decisions, including those related to reproductive status, are taken by the senior members of the family (Pallikadavath et al., 2015). It is worth mentioning that our male attitudinal norms were more prevalent in the Southern compared to Northern regions in India, contrary to Singh et al. (A. Singh et al., 2021) findings of higher prevalence of patriarchal norms in the Northern relative to Southern states. The reason for this discrepancy can be attributed to our selected indicators, which are imperfect/partial measures of patriarchal norms, as they differ substantially—both conceptually and in terms of computation—with comprehensive indicators proposed in the literature for the assessment of patriarchal norms (Gruber and Szołtysek 2016; A. Singh et al., 2021).

In this article, we focus on the effects of gender egalitarian norms as they are embedded in cultural practices and traditions that are persistent in India, and with potential negative influence on gender equality (L. Heise et al., 2019). Here, we focus on five indicators reflecting men’s attitudes towards contraception use, and beliefs towards sexual practices and personal control of their wife. We found persistent attitudes that contraception is women’s business and the belief that those who use it become promiscuous. Although less prevalent nationally and across regions, we also examined beliefs that men have the right to restrict financial support, to have promiscuous behaviors, or even exercise domestic violence if wives refused to have sex. On the other hand, our results indicate that an increasing proportion of women are getting married at older ages, but still falling behind in educational achievement and in the job market, nationally and across regions (IIPS and ICF 2017), perhaps also reflective of the pervasive influence of gender norms.

All the men’s attitudinal indicators examined in this study were negatively associated with contraceptive use among married women with any demand for family planning. Our results of negative effects of men’s attitudinal norms are consistent with previous studies on the effects of domestic violence or male control in India and other countries (Silverman et al., 2008; Mshweshwe 2020; Mshweshwe 2020; Rivera and Scholar 2020; Sikweyiya et al., 2020). The results remained consistent when we only included modern methods, as most women in India rely on these methods. Moreover, when we excluded female sterilization, the effect of men’s attitudinal norms became even stronger, perhaps reflecting the larger autonomy gained by women when they use sterilization (Pallikadavath et al., 2015). This may also relate to the belief that women using contraceptives become promiscuous, as this turned highly significant among modern contraceptive users who did not rely on sterilization and may retain even more control over their contraceptive and fertility preferences. Furthermore, our baseline model indicated a high likelihood of uneducated women using contraception (McNay, Arokiasamy, and Cassen 2003), but the effect no longer held after excluding female sterilization. This is probably related to the fact that uneducated or poor women may be highly influenced by cultural norms to accept this contraceptive method and are relatively less aware of or have lower access to alternative methods of contraception, but further research is also needed.

Our findings of negative influence of men’s attitudinal norms on contraceptive use were robust to women’s empowerment indicators. Our further analysis of contextual effects indicated that even educated or wealthier women in communities with high prevalence of men’s attitudinal norms were less likely to satisfy their demand for contraception. Regarding adult marriage, the results seem contradictory because in regions with high prevalence of men’s attitudinal norms, women who married as adults were less likely to use contraception. This result is, however, consistent with findings from other studies where child marriage is associated with higher contraceptive use and fertility outcomes, including reduced odds of child birth in the first year of marriage, higher lifetime fertility, more terminated pregnancies, and childbirth (Yaya, Odusina, and Bishwajit 2019; S. K. Singh et al., 2019). A plausible explanation for this phenomenon associated with child marriage could be attributed to the attainment of the desired number of children at an earlier age given their early union and higher fertility (Raj et al., 2009; Godha, Hotchkiss, and Gage 2013; Yaya, Odusina, and Bishwajit 2019), which is also consistent with our results of high parity Indian women who had children at very young ages and were more likely to use contraception for limiting childbearing.

Finally, we found that the only indicator of women’s empowerment that was protective to men’s attitudinal norms was employment status, as the probability of contraceptive use among employed women remained high and unchanged even in communities with stronger attitudinal norms. These results indicate that gender restrictive norms are difficult to overcome in specific contexts. Previous studies are in line with this finding; for instance, Bukar et al. (2013) concludes that men still exert a profound influence on contraception in Nigeria, where the level of education had no effect on the independent use of contraceptive methods and even more highly educated women believe that men should influence the contraceptive method of choice. Interestingly, research in Nigeria has also shown that intimate partner violence is lower for working women in communities where women’s labor force participation is normative compared to communities where it is not common for women to work outside the home (Weber et al., 2019). Thus, employment as a mean to empowerment may increase risks of harm for those who challenge restrictive gender norms. Our findings further suggest that employment may play a central role in efforts intended to transform gender roles and norms by encouraging increases in women’s empowerment. However, it is worth mentioning that 85% of rural women in India are engaged in agriculture and they produce about 60–80% of food, yet only around 13% own land and some work on subsistence agriculture or without receiving any payment (Oxfam India 2018). Thus, approaches to prevent, monitor and mitigate harm to women need to be implemented alongside employment promotion as a means to empower women, especially where restrictive norms are strong and women’s labor force participation is low. It is important for gender transformative programs to be inclusive of men and to jointly address gender egalitarian norms. Successful programs should be holistic, address multiple sectors and shareholders, take multiple approaches, and can be initiated by leveraging through more community-based and programmatic efforts (Gupta et al., 2019; Heymann et al., 2019).

### Shortcomings and Limitations

This research has some limitations. First, because of the cross-sectional nature of data, we are unable to make any causal assessment from our findings. Second, our assessment of men’s attitudinal norms or women’s empowerment is an approximation based on the aggregation of individual attitudes/beliefs, or women’s achievements, respectively. Third, a small proportion of women in India are currently employed—mostly in the agricultural sector—and a complete assessment of all women empowerment indicators was only possible with a reduced sample of women. Fourth, our met/unmet need indicators may not be able to capture the potential impact of social resistance, insufficient information/access to methods, and concerns regarding side effects and health impact. Fifth, we did not consider for this study the supply side of contraception uptake mainly because the lack of proper data on access and utilization, a problem recognized in the literature and with potential influence on our results.

### Conclusions and Future Directions

This study provides evidence that men’s attitudinal norms were associated with a lower demand satisfied for contraception. Our assessment is based on statistically robust multilevel associations from five different indicators reflecting aggregate attitudes or beliefs of individual males towards contraceptive use, gender equality and personal control. More importantly, it appears that men’s attitudinal norms dominate women’s empowerment regarding family planning choices, except for women in the labor force who were more likely to satisfy their demand for contraception, even in communities with strong restrictive norms. This is suggestive that programs should address both sets of norms simultaneously, considering the whole ecosystem in which men and women interact, and where addressing women’s empowerment without the consideration of the role of men is not sufficient, and may possibly lead to harm. Our study sheds new light on the importance of cultural factors, but it was limited in its scope and further research is needed. For example, more research is needed to explain the mechanisms of the protective effects of job status on contraceptive uptake in the presence of strong gender restrictive norms. In addition, the role of female sterilization, the dominant method of contraception in India, in the stagnation of met need for family planning requires further study, as it may potentially be inhibiting the use of other forms of contraception across regions, and cultural norms around sterilization may be dominating women’s reproductive autonomy and holding women back from realizing the benefits of broader empowerment initiatives. Future research should consider the supply side of the problem, which may hinder women’s access to contraception method of choice, even when they have the knowledge and empowerment to decide on their reproductive behavior and family planning decisions. The impact of differences in gender egalitarian attitudes (e.g., exploring male and female attitudes and the differences between then) and family planning are also important to explore in future research.

## Data Availability

The raw data supporting the conclusions of this article will be made available by the authors, without undue reservation.

## Footnotes

1 Patriarchy definition, according to the Oxford English Dictionary.

2 We are aware that this indicator may not be ideal in certain contexts due to the complex interplay between paid and unpaid work and levels of employment and autonomy. In our sample, 80% of women reported receiving paid for their work, and our results remain robust after restricting the sample to this group of women for sensitivity analysis (see Materials and Methods).

3 The number 4 in NFHS-4 refers to the fourth wave of the survey. Previous waves were conducted in 1992–93, 1998–99, and 2005–06, respectively.

4 Total demand for family planning is defined as the sum of contraception use and unmet need. Women with unmet need for contraception desire limiting or spacing child bearing but are not using any contraceptive method.

5 In NFHS-4, “country specific” are also listed in both traditional/folkloric and modern methods, but the description of the specific method is not provided.

6 In NFHS-4, currently employed refers to “respondents who were employed in the 7 days before the survey. Includes respondents who did not work in the past 7 days but who are regularly employed and were absent from work for leave, illness, vacation, or any other such reasons” (IIPS and ICF 2017).

7 An even division of the distribution of a variable into five groups.

8 The composition of regions is as follows. Central: 1) Chhattisgarh, 2) Madhya Pradesh, 3) Uttar Pradesh; East: 4) Bihar, 5) Jharkhand, 6) Odisha, 7) West Bengal; North: 8) Chandigarh*, 9) Delhi*, 10) Haryana, 11) Himachal Pradesh, 12) Jammu and Kashmir*, 13) Punjab, 14) Rajasthan, 15) Uttarakhand; Northeast: 16) Arunachal Pradesh, 17) Assam, 18) Manipur, 19) Meghalaya, 20) Mizoram, 21) Nagaland, 22) Sikkim, 23) Tripura; South: 24) Andaman and Nicobar Islands*, 25) Andhra Pradesh, 26) Karnataka, 27) Kerala, 28) Lakshadweep*, 29) Puducherry*, 30) Tamil Nadu, 31) Telangana; and West: 32) Dadra and Nagar Haveli*, 33) Daman and Diu, 34) Goa, 35) Gujarat, 36) Maharashtra. (* Union Territories).
